# College and Hospital Notes

**Published:** 1909-03

**Authors:** 


### COLLEGE AND HOSPITAL NOTES

Providence Retreat, of Buffalo, has just published its thirty-seventh annual report, which is for the year 1908. The Sisters in charge of this hospital for the insane announce the opening of a new modern, commodious and absolutely fire-proof structure. The building is in the form of an oblong, with a central hall and east and west wings. The central hall is completely separated from the rest of the building and is fitted with separate rooms, twelve in number, appropriately furnished with private bath, affording absolute seclusion and quietude and adapted for the care and treatment of the most exacting and refined patients. The wings are devoted to the care and treatment of mental diseases generally. The building is heated and ventilated by the latest improved methods and lighted by electricity throughout.

The Medical Department of Western Reserve University was one of the pioneers in demanding some college work for entrance. In 1898 it announced that, beginning in October, 1901, the completion of the junior year would be required for entrance. In the eight classes which have entered since 1901, an average of 86 per cent. of the matriculates have either held a bachelor's degree on entering or have obtained it at the end of the first medical year.

In May, 1908, the Faculty unanimously voted to recommend a further advance in entrance requirements to the point of requiring a degree for unconditional entrance, but to admit conditionally a man who had completed the junior year in a standard college, (conditioned on the degree being granted by the college from which he had come before his entering the junior year in this medical department). In November, 1908, this vote was unanimously reaffirmed, and on December 17, 1908, the Board of Trustees of Western Reserve University voted that beginning with the academic year 1910-11 (i. e., in October, 1910), the further requirements for entrance to the Medical Department of Western Reserve University shall be in force.

---

Dr. Kenerson's cottage is at 115 Park street, Buffalo, N. Y. Medical, surgical and confinement cases, but no contagious diseases. Privacy and freedom of a home; conveniences, care and diet of a hospital. The patient's own physician and private nurse if desired. Rates, $15.00 to $30.00 per week. Inspection invited. Telephone (not in the book) Frontier 13083.

